# DNA Nanoflower LYTACs Enable Efficient VEGF Degradation and Verteporfin Loading for Combined Therapy of Wet Age‐Related Macular Degeneration

**DOI:** 10.1002/advs.202515852

**Published:** 2026-01-28

**Authors:** Mengxuan Li, Yan Yue, Sijin Wu, Xinyu He, Jiayi Song, Shuwen Ma, Haokun Zhang, Chenyu Xu, Song Chen, Yanming Huang, Songbo Xie, Hua Yan

**Affiliations:** ^1^ Department of Ophthalmology Tianjin Key Laboratory of Ocular Trauma Ministry of Education International Joint Laboratory of Ocular Diseases Tianjin Medical University General Hospital Tianjin China; ^2^ Wisdom Lake Academy of Pharmacy Xi'an Jiaotong‐Liverpool University Suzhou China; ^3^ School of Medicine Nankai University Tianjin China; ^4^ School of Medicine Xiamen Eye Center and Eye Institute of Xiamen University Xiamen Fujian China; ^5^ The Province and Ministry Co‐Sponsored Collaborative Innovation Center for Medical Epigenetics Laboratory of Molecular Ophthalmology Tianjin Medical University Tianjin China

**Keywords:** age‐related macular degeneration, DNA nanoflower, LYTAC, photodynamic therapy, VEGF

## Abstract

Wet age‐related macular degeneration (wAMD), characterized by pathological choroidal neovascularization (CNV), is a leading cause of irreversible vision loss in the elderly. The current standard treatment—anti‐vascular endothelial growth factor (VEGF) therapy—effectively manages neovascularization in many patients. However, some experience suboptimal responses, and frequent intravitreal injections raise safety concerns. Photodynamic therapy is another effective option for treating wAMD, but it can lead to an increase in reactive VEGF after the procedure, resulting in CNV recurrence. In response to these challenges, we propose an integrated approach that combines a DNA nanoflower VEGF degrader with photodynamic therapy. The DNA nanoflower consists of numerous aptamer‐based lysosome‐targeted chimaera (LYTAC) units, which drive extracellular VEGF in the lesion area to the lysosome for degradation. Simultaneously, the DNA nanoflower acts as a carrier for verteporfin (VER), a clinically used photosensitizer. The resulting nanoflower, named NF@VER, generates reactive oxygen species under near‐infrared light to induce endothelial cell death. These combined effects on endothelial cells effectively block VEGF‐induced CNV in vivo, without causing noticeable side effects. Overall, this innovative approach presents a precise and effective strategy for treating wAMD, reducing the risk of VEGF reactivation‐induced CNV recurrence, and minimizing the systemic side effects associated with photodynamic therapy.

## Graphical abstract

An integrated approach that combines a DNA nanoflower VEGF degrader with photodynamic therapy is developed for the treatment of wet age‐related macular degeneration, which reduces the risk of VEGF reactivation‐induced CNV recurrence and minimizes the systemic side effects associated with photodynamic therapy.

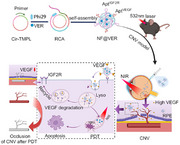

## 1 Introduction

By 2040, approximately 288 million people worldwide are expected to suffer from age‐related macular degeneration (AMD), the leading cause of significant vision impairment in older adults [1]. AMD has two types: dry AMD and wet AMD (wAMD) [2], with the latter causing severe and irreversible vision loss in over 90% of cases [3, 4]. wAMD is characterized by choroidal neovascularization (CNV) in the subretinal region, causing concomitant retinal damage [5, 6]. The clinical treatments for CNV include antiangiogenic therapy [7], thermal laser treatment [8], and photodynamic therapy (PDT) [9], all of which aim to slow the progression of neovascularization. Remarkably, CNV is primarily triggered by vascular endothelial growth factor (VEGF), which is upregulated in response to stimuli such as oxidative stress and complement activation [10]. Despite the remarkable clinical outcomes of intravitreal injection of anti‐VEGF agents (e.g., aflibercept, bevacizumab, and ranibizumab), limited effectiveness for some patients and the requirement of frequent intraocular injections present a challenge [11, 12, 13, 14].

PDT with light‐activated photosensitizing agents, such as verteporfin (VER), is an effective treatment option [4]. By generating ROS and free radicals, it damages 0endothelial cells, thereby leading to the occlusion of new blood vessels [9, 15]. However, systemic administration of VER may cause some side effects, including injection site reactions, infusion‐related back pain, nausea, photosensitivity reactions, asthenia, and increased cholesterol levels [15]. Furthermore, PDT may impact the choriocapillaris bed surrounding the pathological CNV lesion, resulting in hypoxia that promotes VEGF expression and further CNV recurrence [4, 16]. Recent studies show that combining PDT with anti‐VEGF therapy reduces the need for single anti‐VEGF injections and produces better outcomes than single therapy [17, 18, 19].

Lysosome‐targeting chimeras (LYTACs) are an innovative strategy that induces the degradation of extracellular and membrane proteins via the endosomal‐lysosomal pathway [20]. LYTACs comprised a warhead designed to specifically bind to the protein of interest (POI) [21], coupled with another warhead that binds to the lysosome‐shuttling receptor, which includes insulin‐like growth factor 2 receptor (IGF2R) [22], asialoglycoprotein receptor (ASGPR) [23], transferrin receptor (TfR) [24], folate receptor α (FRα) [25], etc. Recent advancements have demonstrated that aptamer‐based LYTACs hold great promise due to their advantages in facile synthesis, high tissue penetration, and multivalent manipulation potential [26, 27, 28]. However, the relatively low degradation efficiency of monovalent aptamer‐based LYTACs limits their translational potential. To improve degradation efficiency, we designed a DNA nanoflower (NF)‐based multivalent LYTAC that contains numerous aptamers targeting VEGF and IGF2R, respectively [29, 30]. Concurrently, the photosensitizing agent VER was encapsulated by the NF‐based LYTAC, allowing for combination therapy for wAMD.

## 2 Results

### 2.1 Construction and Characterization of NF@VER

**FIGURE 1:**
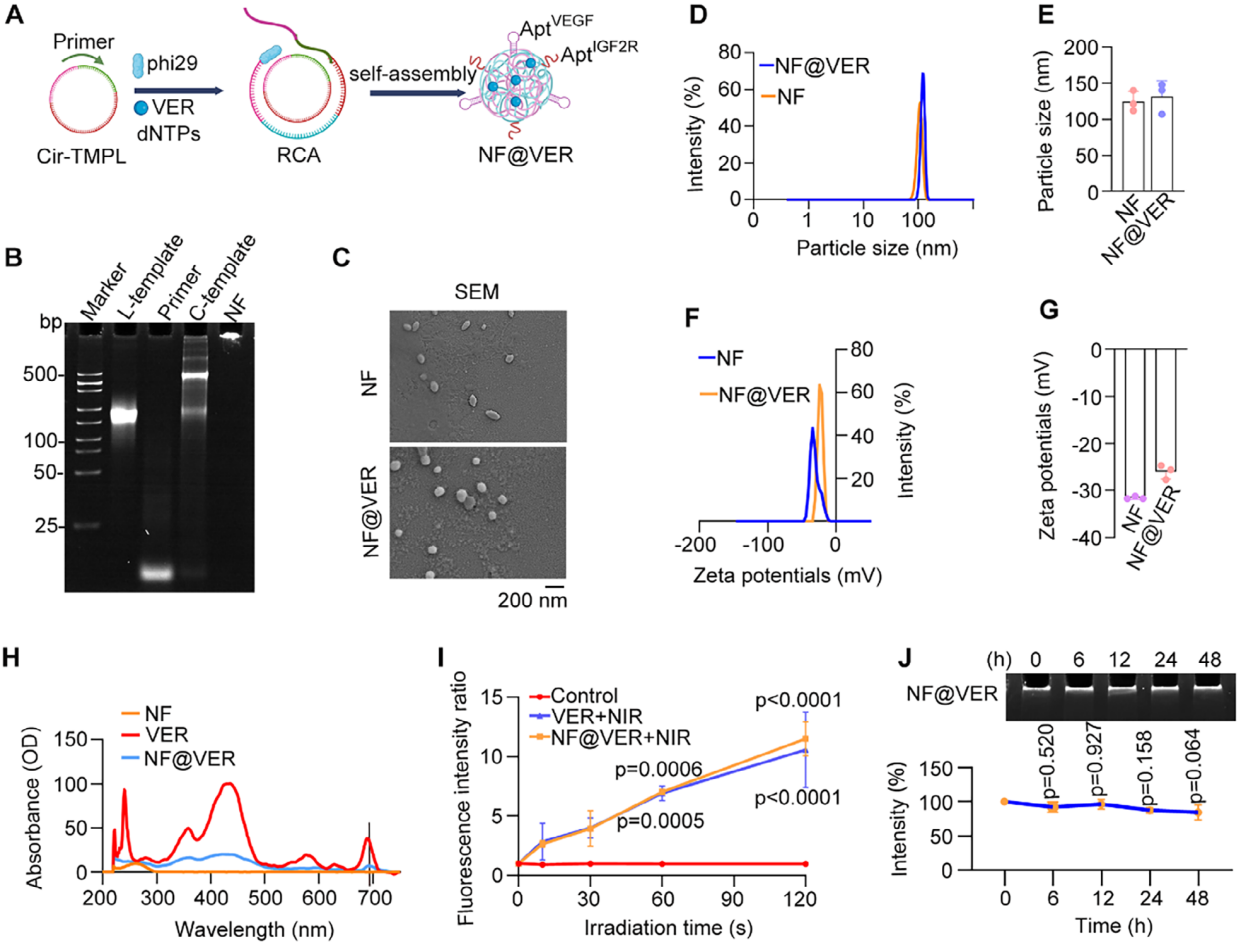
Characterization of NF@VER. (A) Schematic of the NF@VER construction via rolling cycle amplification (RCA) (Created with BioRender.com). (B) Native polyacrylamide gel analysis of NF. (C) SEM images of NF and NF@VER. (D, E) The particle size of NF and NF@VER (*n* = 3). (F, G) The zeta potentials of NF and NF@VER. (H) The ultraviolet and visible spectrum (UV–vis) absorption spectra of NF, VER, and NF@VER. (I) The ROS fluorescence signals of 500 nm VER and 500 nm NF@VER exposed to 690 nm NIR irradiation were detected using the singlet oxygen sensor green (SOSG) probe (*n* = 3). (J) Native polyacrylamide gel analysis of the stability of NF@VER in 10% serum (*n* = 3). Statistical analyses were performed using Two‐way ANOVA followed by Dunnett's multiple comparisons tests (I) and One‐way ANOVA followed by Tukey's multiple comparisons tests (J). Data are presented as mean ± SD.

To enhance internalization efficiency and facilitate combination therapy, we utilized the roll cycle amplification (RCA) method to create multiple aptamer‐based LYTAC‐containing NF loaded with VER, termed NF@VER hereafter (Figure 1A). The circular template for NF includes complementary sequences of VEGF‐targeted aptamer and IGF2R‐targeted aptamer (Table S1). Native PAGE gel electrophoresis indicated the successful construction of the NF (Figure 1B). SEM images and zeta sizer analysis confirmed the success of NF generation and that VER loading did not significantly increase the particle size, with the particle size shifted only slightly from 124.4 ± 8.3 to 131.7 ± 12.5 nm (Figure 1C–E). Zeta potential of NF and NF@VER was ‐31.5 ± 0.2 mV and ‐26.0 ± 0.9 mV, respectively, indicating the relative stability of these nanoparticles (Figure 1F,G). The encapsulation efficiency (EE%) of VER was 33.79 ± 3.14%, and the loading capacity was 3.9 ± 0.36% (Figure S1A). Spectral analysis revealed that VER was effectively incorporated into the NF. The 5 nm redshift in the Q‐band peak of NF@VER (695 nm) versus free VER (690 nm) is attributed to non‐covalent assembly, primarily through π‐π intercalation with DNA bases (Figure 1H).

To elucidate the molecular mechanism of VER integration into DNA nanostructures, we conducted molecular dynamics (MD) simulations of the two VER isomers (VER‐C & VER‐D) in conjunction with single‐stranded DNA fragments. Both isomers of VER reached dynamic equilibrium and formed a stable binding conformation within the DNA pocket (Figure S2A). Analysis of equilibrium trajectories (50–200 ns) revealed that VER‐C integrates into the helix via synergistic hydrogen bonding and π‐π stacking (Figure S2B,C). In contrast, VER‐D binding is stabilized by a hydrogen bond network complemented by a unique two‐site π‐π stacking mechanism (Figure S2D,E). Moreover, the sustained proximity between DG77 and both isomers throughout the simulations suggests that this nucleotide serves as a “key anchor” for maintaining ligand stability (Figure S2F,G). Together, these results confirm that VER can be stably encapsulated in DNA structures through non‐covalent interactions.

We further monitored the production of reactive oxygen species (ROS) under 690 nm NIR irradiation (100 mW/cm^2^) using a singlet oxygen sensor green (SOSG). NF@VER released comparable fluorescence intensity to that of free VER under 690 nm NIR irradiation (Figure 1I), confirming the successful loading. Notably, serum stability assays demonstrated that no significant degradation of NF@VER was observed even at 48 h (Figure 1J), highlighting the stability of the NF@VER nanoparticle.

### 2.2 NF Effectively Drives VEGF to Lysosomes for Degradation

**FIGURE 2:**
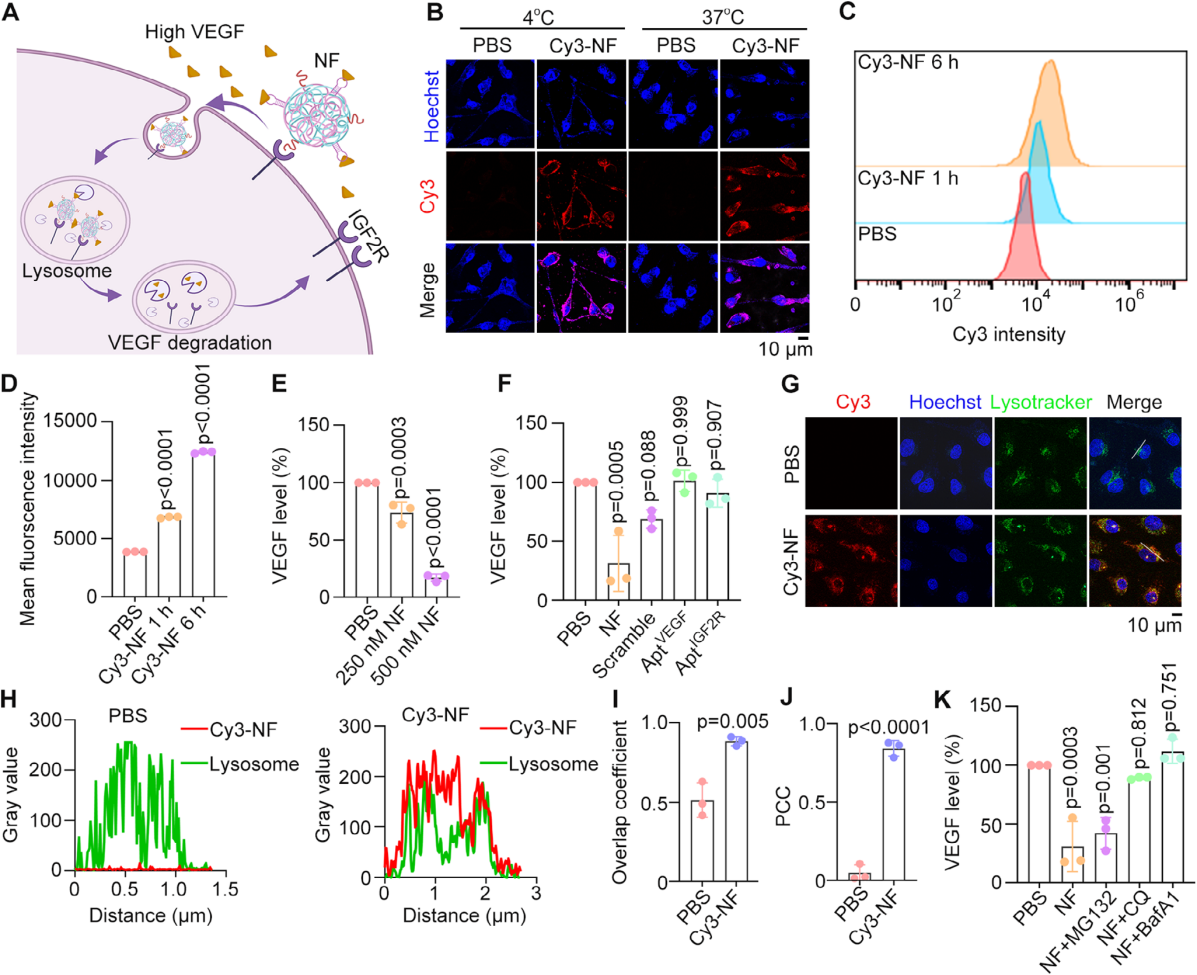
NF drives VEGF to the lysosome for degradation. (A) Schematic of the NF degrading VEGF through the lysosomal pathway (Created with BioRender.com). (B) Fluorescence images of the uptake of 500 nm Cy3‐labeled NF at 4°C or 37°C. (C, D) Flow cytometry detection (C) and quantification (D) of the internalization efficacy of 500 nm Cy3‐labeled NF (*n* = 3). (E, F) ELISA assays to analyze the VEGF level in EA. hy926 cells treated with the indicated concentrations of NF (E) or various controls (F) (*n* = 3). (G‐J) Fluorescence images of EA. hy926 cells treated with PBS or 500 nm Cy3‐labeled NF, followed by lysotracker and Hoechst staining (G). The colocalization of lysosomes and NF (H), overlap coefficient (I), and Pearson correlation coefficient (PCC) of colocalization (J) were determined (*n* = 3). (K) The ELISA assay is used to analyze the VEGF level in EA. hy926 cells were treated with PBS or 500 nm NF together with MG132 (10 µm), chloroquine (CQ, 10 µm), and BafA1 (100 nm) for 12 h (*n* = 3). Statistical analyses were performed using One‐way ANOVA followed by Tukey's multiple comparisons tests (D, E, F, K) and a non‐paired two‐tailed Student's *t*‐test (I, J). Data are presented as mean ± SD.

The NF consists of many LYTAC functional units capable of degrading VEGF, in which the IGF2R‐targeted aptamer binds to the IGF2R on the cell membrane, facilitating the endocytosis of VEGF for lysosomal degradation (Figure 2A). To evaluate the uptake efficacy, NF were added to the culture medium of EA. hy926 endothelial cells. Initially, the temperature was set to 4°C to inhibit membrane fluidity, allowing for better observation of Cy3‐labelled NF binding to surface IGF2R. However, when the temperature was adjusted to 37°C for 1 h, significant uptake of Cy3‐labelled NF was observed (Figure 2B). Flow cytometry results demonstrated an increased uptake of Cy3‐labeled NF over time (Figure 2C,D). Next, we investigated the effect of NF on VEGF degradation. Enzyme‐linked immunosorbent assays (ELISA) revealed a significant decrease in VEGF levels following NF treatment in a dose‐dependent manner (Figure 2E). In contrast, treatments with the VEGF aptamer, IGF2R aptamer, or scrambled NF control did not lead to a reduction in VEGF levels (Figure 2F). Additionally, the inhibition of downstream pathways following NF‐mediated VEGF degradation was evaluated. This effect was reflected in a marked reduction in phosphorylated AKT levels upon NF treatment (Figure S3).

Next, we analyzed whether the NF facilitates the transport of VEGF to lysosomes for degradation. Fluorescence microscopy images showed colocalization of NF with lysosomes (Figure 2G). The overlap coefficient and Pearson correlation coefficient indicated a significant correlation between NF and lysosomes, as shown in the colocalization curve (Figure 2H–J). Furthermore, NF‐mediated VEGF degradation was remarkably suppressed by chloroquine (CQ) and bafilomycin A1 (BafA1), the autophagy‐lysosomal inhibitors, but not the proteasome inhibitor MG132 (Figure 2K). Overall, these findings demonstrate that the NF promotes VEGF degradation through the lysosomal pathway and effectively inhibits the phosphorylation of downstream proteins of VEGF.

### 2.3 NF@VER Induces ROS Production and Endothelial Cell Death under NIR Irradiation

**FIGURE 3:**
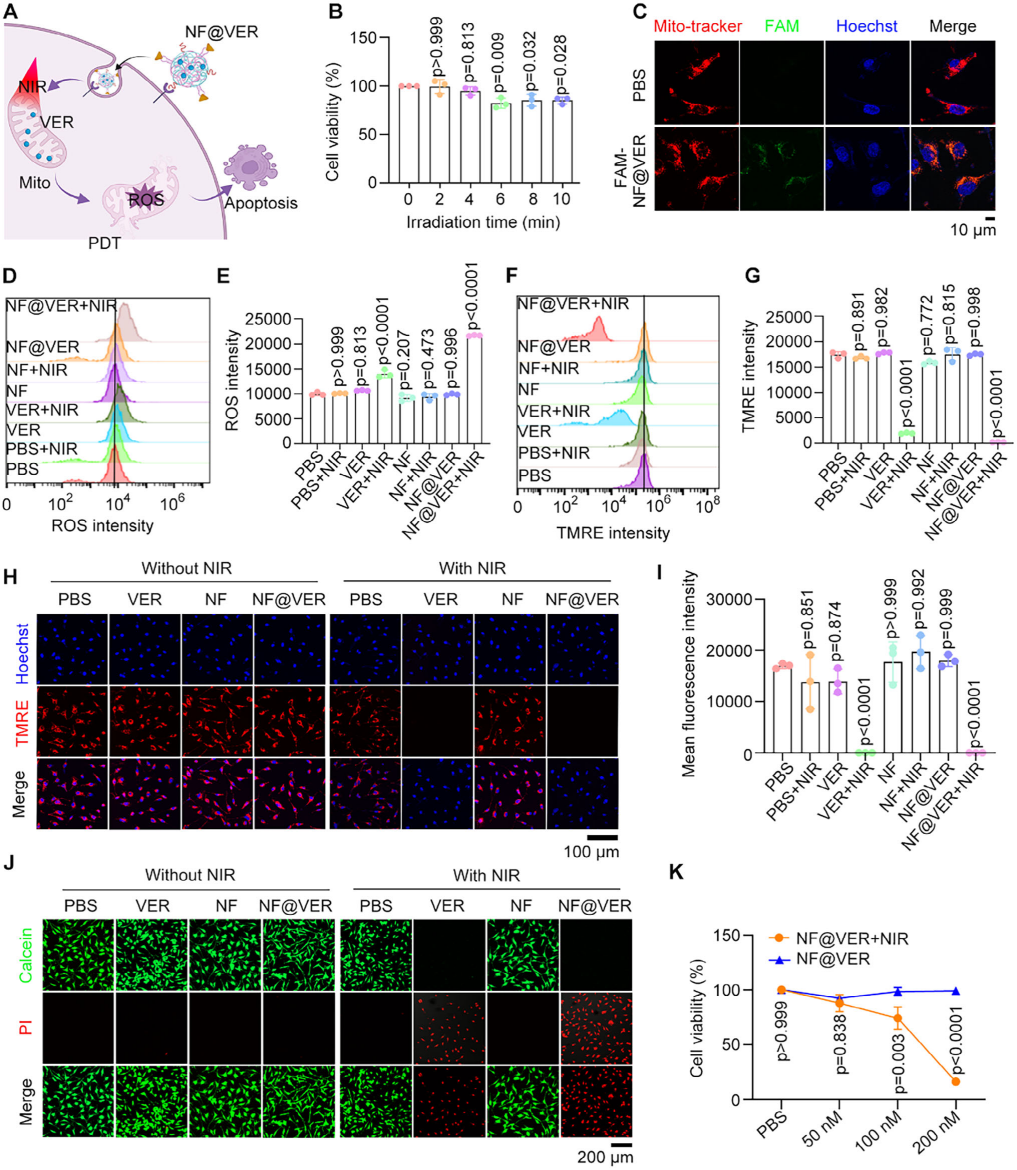
NF@VER enables ROS‐induced endothelial cell death under NIR irradiation. (A) Schematic of the NF@VER inducing cell death under NIR irradiation (Created with BioRender.com). (B) Cell viability determination of EA. hy926 cells under the indicated time of NIR irradiation (100 mW/cm^2^, *n* = 3). (C) Fluorescence images of EA. hy926 cells were treated with PBS or 500 nm FAM‐labeled NF@VER, followed by Mito‐tracker and Hoechst staining. (D, E) Flow cytometry detection (D) and quantification (E) of intracellular ROS generation by 500 nm VER and 500 nm NF@VER with or without NIR irradiation (*n* = 3). (F‐I) Flow cytometry detection (F) and fluorescence images (H) of EA. hy926 cells treated with PBS, 500 nm VER, 500 nm NF, or 500 nm NF@VER with or without NIR irradiation (100 mW/cm^2^, 4 min), followed by mitochondrial membrane potential detection using the TMRE probe. The TMRE signals were quantified (*n* = 3). (J) Fluorescence images of EA. hy926 cells treated with PBS, 500 nm VER, 500 nm NF, or 500 nm NF@VER with or without NIR irradiation, followed by live and dead cell staining with Calcein‐AM/PI. (K) Cell viability of EA. hy926 cells treated with various concentrations of NF@VER with or without NIR irradiation (*n* = 3). Statistical analyses were performed using One‐way ANOVA followed by Tukey's multiple comparisons test (B, E, G, I) and Two‐way ANOVA followed by Šídák's multiple comparisons test (K). Data are presented as mean ± SD.

Given that VER can produce ROS to induce vascular endothelial cell death and blood vessel occlusion under NIR irradiation [31]. We next investigated the effects of NF@VER on ROS‐mediated endothelial cell death (Figure 3A). The cytotoxicity of NIR exposure on EA. hy926 cells were evaluated. The CCK‐8 assay results showed that irradiation times of 2 and 4 min had little effect on cell viability (Figure 3B). Therefore, NIR irradiation of 4 min was chosen for subsequent experiments. The minimum effective concentration of VER was found to be 500 nm, at which cells were killed under NIR light but were viable in its absence (Figure S4A). Therefore, an equivalent amount of NF@VER (500 nm VER) was used for the subsequent experiments.

Fluorescence images show that NF@VER was efficiently internalized into endothelial cells and colocalized with the mitochondria (Figure 3C and Figure S4B–E). Flow cytometry analysis confirmed the efficient uptake of NF@VER by ARPE19 cells (Figure S4F,G). However, no obvious effects on cell activity were observed (Figure S4H). The ROS generation capability of NF@VER was assessed using a 2, 7'‐dichlorofluorescein diacetate (DCFH‐DA) probe. Cells treated with VER or NF@VER displayed intense fluorescence signals after NIR irradiation, indicative of ROS production. In contrast, negligible DCFH‐DA fluorescence was observed in the PBS or NF treatment group according to flow cytometry results (Figure 3D,E). In addition, NF@VER did not increase intracellular ROS levels without NIR irradiation (Figure S4I,J). As ROS usually leads to mitochondrial damage [32], we examined the effects of NF@VER on mitochondrial membrane potential. The fluorescence signals emitted by tetramethylrhodamine ethyl ester (TMRE), an indicator of mitochondrial membrane potential, were significantly reduced in VER or NF@VER treatment under NIR irradiation (Figure 3F–I). Furthermore, live‐dead cell staining indicated that VER or NF@VER effectively induced cell death under NIR (Figure 3J). CCK‐8 assays demonstrated that although NF@VER did not affect cell viability without NIR irradiation, its dose‐dependent cell‐killing was activated by NIR (Figure 3K). These results confirm that NF@VER is inert without NIR irradiation, making it a promising candidate for treating CNV‐related diseases.

### 2.4 NF@VER Exhibits Profound Anti‐angiogenic Activity In Vitro

**FIGURE 4:**
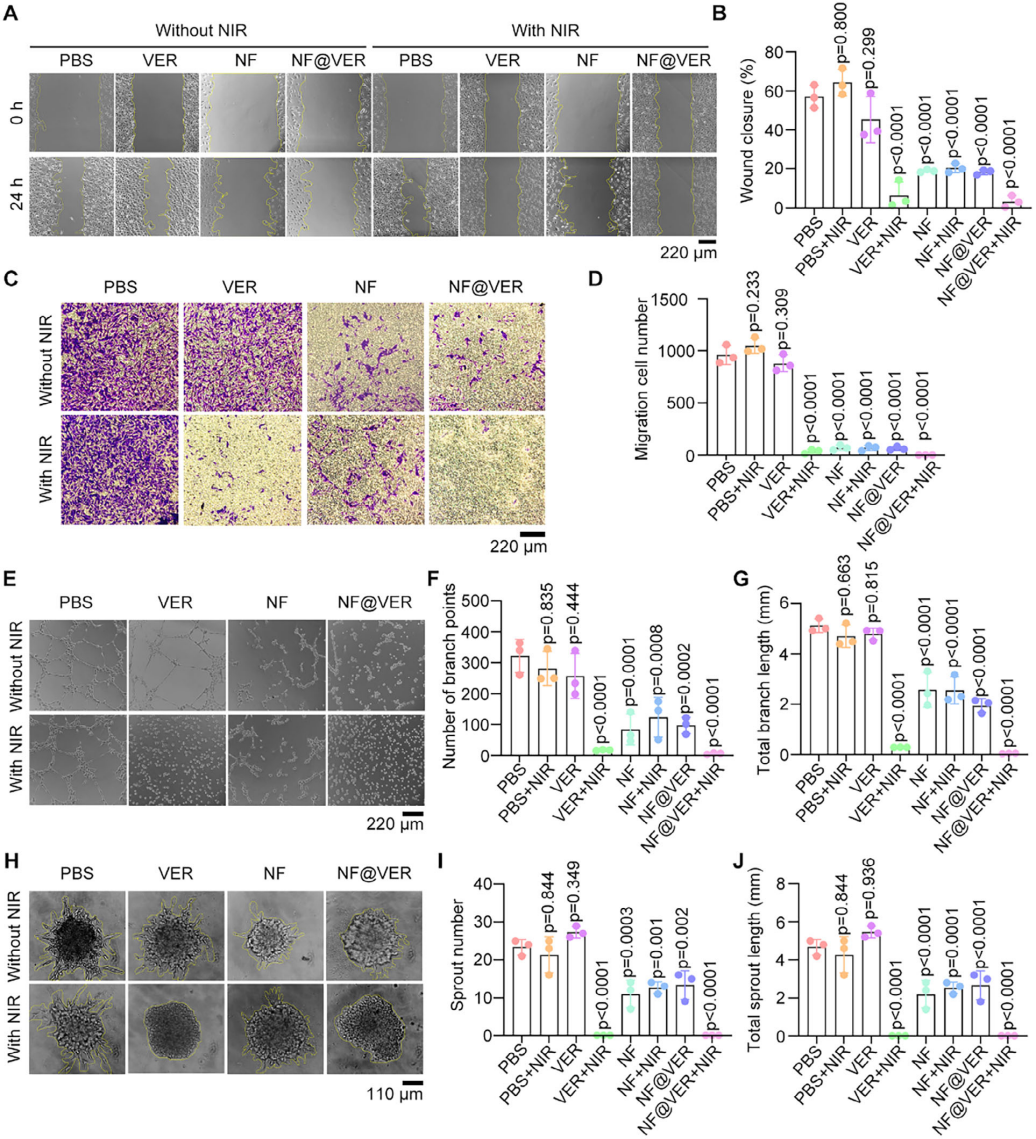
NF@VER suppresses VEGF‐dependent angiogenesis in vitro. (A, B) EA. hy926 cells were treated with PBS, 500 nm VER, 500 nm NF, or 500 nm NF@VER with or without NIR irradiation. The wound healing regions were imaged (A), and the percentage of wound closure was quantified (B) (*n* = 3). (C, D) Representative transwell images of EA. hy926 cells treated with PBS, 500 nm VER, 500 nm NF, or 500 nm NF@VER with or without NIR irradiation (C), and the number of migrating cells was quantified (D) (*n* = 3). (E‐G) Representative tube formation images of EA. hy926 cells treated with PBS, 500 nm VER, 500 nm NF, or 500 nm NF@VER with or without NIR irradiation (E). The number of branch points (F) and the total branch length (G) were quantified (*n* = 3). (H‐J) Representative sprouting images of EA. hy926 cell spheroids treated with PBS, 500 nm VER, 500 nm NF, or 500 nm NF@VER with or without NIR irradiation (H). The sprout numbers (I) and length (J) in the sprout assays were quantified (*n* = 3). Statistical analyses were performed using One‐way ANOVA followed by Tukey's multiple comparisons tests (B, D, F, G, I, J). Data are presented as mean ± SD.

Next, we examined the inhibitory effects of NF@VER on angiogenesis. Since the migration of endothelial cells is essential for the formation of new blood vessels, we initially conducted wound‐healing and transwell assays. VER with NIR irradiation and treatment with NF or NF@VER alone significantly inhibited the migration of endothelial cells, indicating the anti‐angiogenic potential of VEGF degradation combined with PDT. Moreover, NIR irradiation enhanced the anti‐migration ability of NF@VER, confirming the synergistic effects (Figure 4A–D). Consistently, NF or NF@VER treatment markedly inhibited endothelial tube formation, and NIR irradiation aggravated the inhibitory effect of NF@VER, probably because of the combined effects of VER‐mediated PDT and NF‐mediated VEGF exhaustion (Figure 4E–G). Further 3D spherical cell sprouting assays confirmed the profound role of NF@VER in neovascularization (Figure 4H–J). Taken together, NF@VER exerts its anti‐angiogenic action by exhausting VEGF combined with PDT.

### 2.5 NF Suppresses VEGF‐induced Vascular Leakage In vivo

The increased VEGF is the main cause of the formation of new blood vessels and vascular leakage in the eyes. We then established a VEGF‐induced retinal vascular leakage model to verify the therapeutic effect of NF (Figure S5A). Fundus fluorescein angiography (FFA) revealed a significant fluorescein leakage, indicating the successful model establishment. Both Conbercept and NF effectively suppressed VEGF‐induced vascular leakage (Figure S5B). This finding was further confirmed by FITC‐dextran perfusion assays (Figure S5C,D). In summary, NF significantly inhibits VEGF‐induced retinal vascular leakage in mice.

### 2.6 NF@VER Attenuates the Experimental CNV in vivo

**FIGURE 5:**
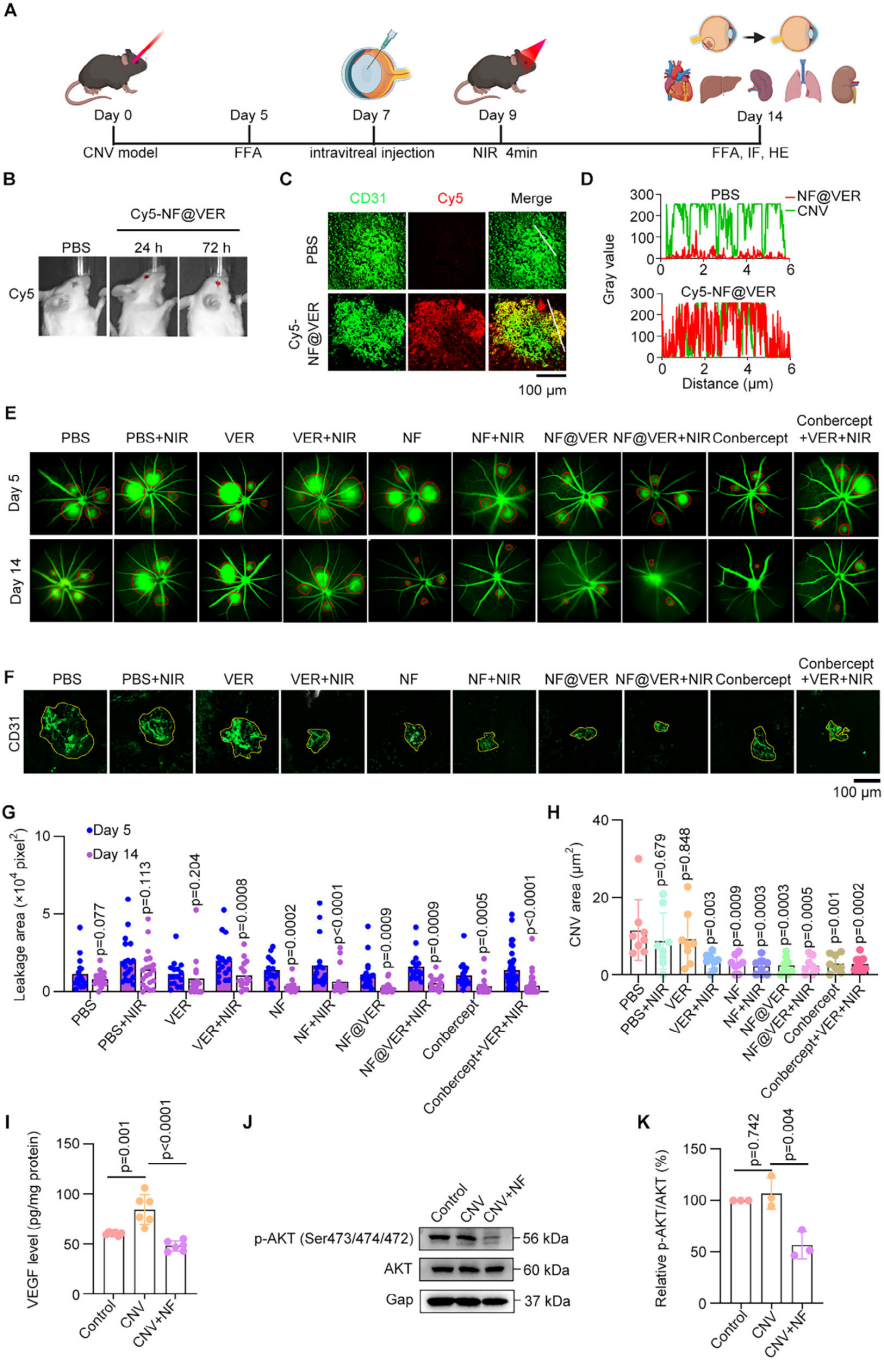
NF@VER attenuates experimental CNV in vivo. (A) Schematic of the animal experiments (Created with BioRender.com). (B) in vivo imaging of intravitreally injected 2 µm Cy5‐labeled NF@VER in CD1 mouse. (C, D) Fluorescence images showing localization of 2 µm NF@VER in the CNV regions. (E, G) Fundus fluorescein angiography (FFA) images (E) and quantifications (G) of laser‐induced CNV mice on Day 5 and Day 14. (F, H) Fluorescence images (F) and quantifications (H) of the choroid‐RPE complex in the CNV area (*n* = 6 mice). (I) ELISA assays to analyze the VEGF levels in the retinal‐choroid complex of CNV mice treated with 2 µm of NF or not (*n* = 6 mice). (J, K) Immunoblots and quantifications of p‐AKT/AKT levels in the retinal‐choroid complex of NF‐treated CNV mice (*n* = 3). Statistical analyses were performed using Multiple paired t‐tests (G), and One‐way ANOVA followed by Tukey's multiple comparisons tests (H, I, K). Data are presented as mean ± SD.

We examined the effect of NF@VER in vivo using a laser‐induced CNV mouse model. After confirmation of the successful modeling using fundus fluorescein angiography (FFA) on Day 5, NF@VER was intravitreally injected on Day 7, followed by NIR irradiation on Day 9 and efficacy and toxicity evaluation on Day 14 (Figure 5A). Cy5‐labeled NF@VER remained visible in the eyes of the mice 72 h post‐injection, suggesting its stability within the ocular environment (Figure 5B). Further immunostaining of the retinal section revealed that Cy5‐labeled NF@VER co‐localized with CD31, an endothelial cell marker, indicating its accumulation in the CNV region (Figure 5C,D). FFA results demonstrated that significant hyperfluorescent neovascular leakage occurred on Day 5, indicative of the successful establishment of the CNV mouse model (Figure 5E). Strikingly, NF@VER treatment resulted in an obvious reduction of the leakage area under NIR irradiation, which is comparable to the therapeutic effect of Conbercept treatment (Figure 5E,G). Immunofluorescence staining of retinal pigment epithelium‐choroid flat mounts revealed that NF@VER with NIR irradiation led to almost complete occlusion of the CNV (Figure 5F,H). Furthermore, NF effectively degraded VEGF and reduced the phosphorylated AKT (Figure 5I–K). These data indicate the therapeutic potential of NF@VER in treating CNV‐related diseases.

### 2.7 The Long‐term Intraocular Safety and Systemic Immunogenicity Examination of NF@VER

**FIGURE 6:**
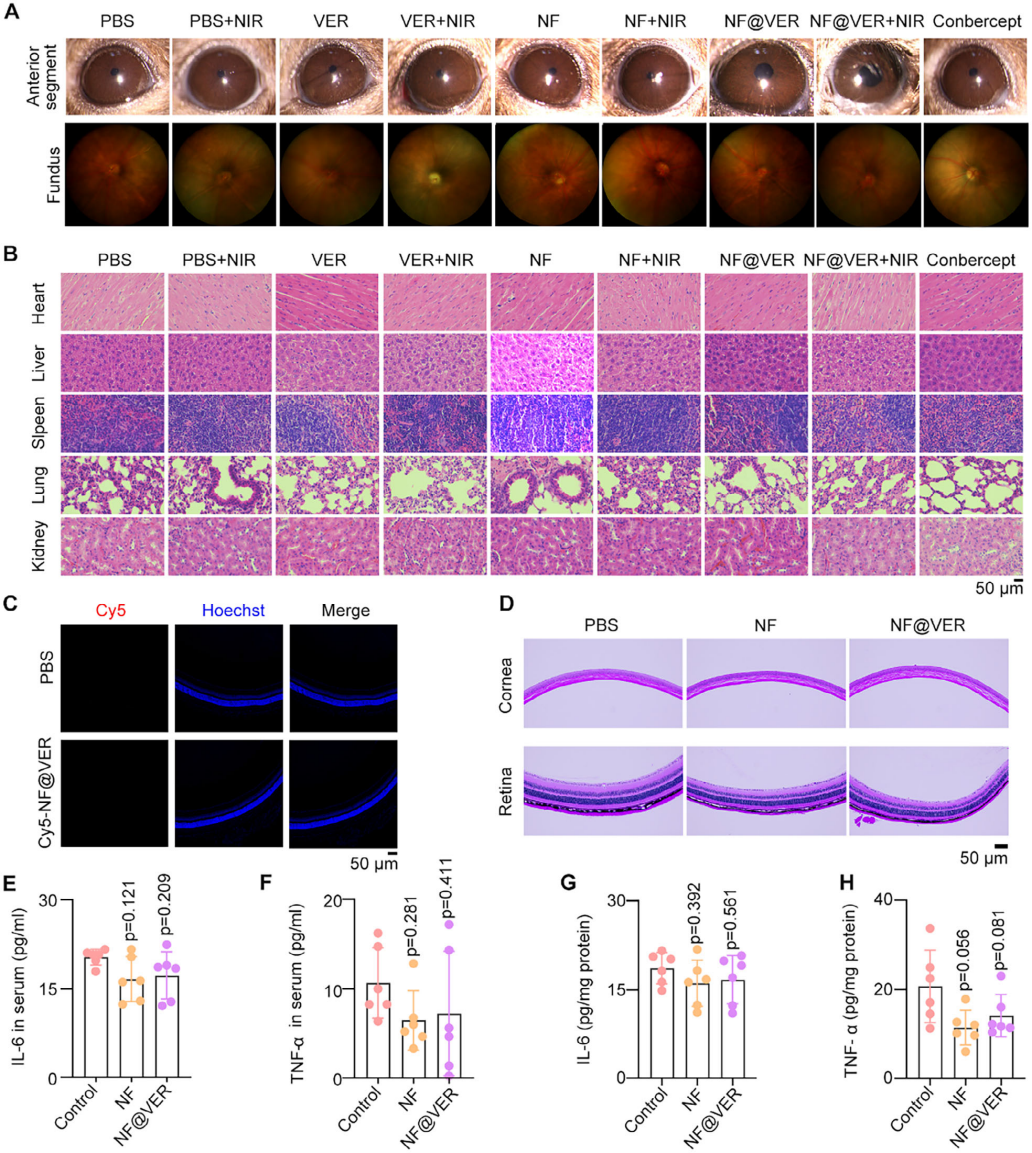
(A) The anterior segment and fundus images of mice eyes after treatment. (B) HE images of heart, liver, spleen, lung, and kidney isolated from the control and treatment CNV mice. (C) The intraocular tissue distribution of 2 µm Cy5‐NF@VER 30 days after intravitreal injection. (D) HE staining of the mouse eyeball tissue after intravitreal injection of 2 µm NF@VER 30 days. (E–H) The levels of inflammatory factors IL‐6 and TNF‐α in the serum and the retinal choroid complex of mice 30 days after intravitreal injection of 2 µm NF, or NF@VER (*n* = 6 mice). Statistical analyses were performed using One‐way ANOVA followed by Tukey's multiple comparisons tests (E, F, G, H). Data are presented as mean ± SD.

Next, we comprehensively evaluated the biosafety of NF@VER. Initial ocular examinations and fundus photography showed that almost no abnormalities in corneal, lens, or retinal tissues were observed in all the groups (Figure 6A). Consistently, H&E staining of major organs revealed no obvious damage after 14 days of treatment (Figure 6B), indicating the systemic safety of NF@VER. We next detected the potential toxicity of NF@VER in a prolonged period. Cy5 signals in retina sections were undetectable on Day 30, indicating the clearance of NF@VER (Figure 6C). No obvious changes were found in HE staining of the ocular tissues (Figure 6D), and the levels of IL‐6 and TNF‐α in the serum and the retinal‐choroid complex were not affected by NF@VER (Figure 6E–H). These data indicate the long‐term safety of NF@VER.

## 3 Discussion

The hydrophobic property of VER and side effects caused by systemic administration have long limited its application [33, 34]. In clinical settings, a relatively high dosage of VER is required, which may cause adverse reactions, such as photosensitive skin damage, throughout the treatment process [35]. To resolve these safety issues, some studies have utilized liposome encapsulation to improve its water solubility and its enrichment in the lesion area [36, 37]. In addition, clinical studies have shown that combining PDT with anti‐VEGF treatment is more effective than either therapy alone and reduces the frequency of anti‐VEGF injections [17]. However, how to lower the side effects during the combination therapy remains unaddressed. In this study, we develop an integrated NF strategy to block the VEGF pathway and load VER for PDT effectively. The hydrophilic NF not only addresses the insolubility issue of hydrophobic photosensitizers but also enhances the targeted delivery efficiency of VER, as demonstrated that locally‐administered NF@VER was largely localized at CNV areas within the eye, probably due to its efficient uptake by the newly formed blood vessels.

Another issue associated with photodynamic therapy is the recurrence of CNV and treatment failure caused by its oxygen‐consuming property, which leads to hypoxia‐induced VEGF upregulation [38]. Our research demonstrates that NF@VER effectively degrades VEGF, which may compromise the VEGF feedback issues of PDT and achieve a comprehensive CNV treatment effect. Indeed, NF@VER demonstrated a superior inhibitory effect over Conbercept treatment on CNV in cell‐based assays. However, only slight synergistic effects between VEGF inhibition and PDT were observed in the laser‐induced CNV mouse model, probably due to that this model primarily represents an acute phase of CNV development over approximately two weeks, which does not recapitulate the clinical long‐term settings of treatment resistance.

In summary, we developed an integrated modality, NF@VER, comprised of multiple aptamer‐based LYTAC units that function as carriers for loading photosensitizers, offering a promising solution for the combined treatment of wAMD.

## 4 Experimental Section

### 4.1 Cell Culture

EA. hy926 human umbilical vein cells (#ZKC1051‐1, RRID: CVCL_3901 EA. hy 926) were purchased from Beijing Zoman Biotechnology. Cells were cultured in Gibco Dulbecco's Modified Eagle medium (DMEM) with 10% fetal bovine serum (FBS) and 1% penicillin‐streptomycin solution at 37°C in a humidified atmosphere containing 5% CO_2_. ARPE19 cells (iCell‐h020, Cellverse, China) were cultured in F12 medium with 10% FBS and 1% penicillin‐streptomycin at 37°C in a humidified atmosphere containing 5% CO_2_. Routine mycoplasma testing was performed to ensure the cells were contamination‐free. EA. hy926 cells were chosen to establish the CNV‐related cell model and investigate the effects of NF@VER on CNV.

### 4.2 Synthesis of NF and NF@VER

The linear DNA template comprising the corresponding sequence of the aptamer of VEGF and IGF2R was sourced from BGI Genomics (Beijing, China). To prepare the circular template, the DNA linear aptamers and primers (Table S1) were heated to 95°C for 5 min and then slowly cooled to room temperature over 3 h. T4 ligase (#10301ES42, YEASEN, Shanghai, China) was subsequently added and incubated overnight, followed by heating at 65°C for 10 min to extinguish T4 ligase. The circular template, dNTPs, and phi29 DNA polymerase (E013‐01B, Novoprotein, Shanghai, China), with or without VER (V873898, Macklin, China), were then amplified at 30°C for 3 h to generate nanoflower structures [39]. To verify the successful assembly of the NF, the reaction mixtures were analyzed on a 10% native polyacrylamide gel electrophoresis (PAGE), stained with Gel Red for DNA visualization. The stability of the NF@VER was tested using a 10% native PAGE after incubation with 10% serum at 37°C for at least 48 h.

### 4.3 Molecular Docking and Simulation

The 3D structure of the single‐stranded DNA fragment involved in the RCA process was generated using the 3dRNA/DNA software package. The structures of the two VER isomers were obtained from the PubChem database and processed with OpenBabel. Molecular docking was performed with AutoDock Vina to predict a favorable initial binding pose. The most probable binding pocket on the DNA was identified, and each VER isomer was docked into this site. The complex with the highest binding affinity was selected as the starting structure for subsequent MD simulations.

All systems were built using the tleap module of AMBER. The DNA was described by the OL15 force field, VER by the General Amber Force Field (GAFF), and water by the TIP3P model. Force field parameters and partial atomic charges for VER were generated using the Antechamber suite. The docked DNA‐VER complex was solvated in a periodic box of TIP3P water with a minimum distance of 12.0 Å between any solute heavy atom and the box edge. Counterions (Na^+^ or Cl^−^) were added to neutralize the system.

All‐atom MD simulations were performed using the pmemd. CUDA engine of AMBER. The protocol involved: (1) Minimization: A multi‐step energy minimization was performed to remove steric clashes, first on the solvent and ions with the solute restrained, followed by an unrestrained minimization of the entire system. (2) Heating and equilibration: The system was heated from 0 to 300 K in the NVT ensemble, then equilibrated in the NPT ensemble until system density and pressure stabilized at 1 atm. (3) Production run: Production runs of 200 ns were conducted for each complex under NPT conditions (300 K, 1 atm). A 2.0 fs time step was used by constraining all bonds involving hydrogen with the SHAKE algorithm. Temperature was maintained using a Langevin thermostat, and pressure was controlled by an isotropic barostat. A 10.0 Å cutoff was applied for non‐bonded interactions, with long‐range electrostatics treated by the Particle Mesh Ewald (PME) method. Trajectories were saved every 40 ps.

Post‐simulation analysis was performed on the trajectories using the cpptraj module of AMBER and the MDAnalysis Python library. System stability was assessed by calculating the RMSD of the ligand's heavy atoms. Key non‐covalent interactions were quantified, including hydrogen bond and π–π stacking, which were identified by monitoring geometric criteria (centroid distance and angle) between the aromatic rings of VER and DNA bases. Based on the interaction analysis, key DNA bases were identified. The minimum heavy‐atom distance between VER and these bases was tracked over time to quantify the proximity and persistence of the association, confirming the stable integration of VER.

### 4.4 NF and NF@VER Characterization

The morphology of the NF and NF@VER was examined using a scanning electron microscope (Gemini 300, Zeiss, Germany), and the size was measured by dynamic light scattering (DLS) with a Zetasizer Nano ZS instrument (Nano ZS90, Malvern, England). To determine the loading of VER, the UV–vis absorption spectra were measured using a Nanodrop 2000 (Thermo Fisher, MA, USA). VER encapsulation efficiency and VER‐loading capacity were determined by mass spectrometry (LTQ‐Orbitrap, Thermo Fisher, USA).

### 4.5 ROS Detection

Free VER and NF@VER were incubated with SOSG (#A0415E, Meilunbio, Dalian, China) to achieve final concentrations of VER at 500 nm and SOSG at 2 µm in double‐distilled water. The solution was irradiated with a 690 nm laser at 100 mW cm^−2^ for different durations. The SOSG solution without the photosensitizer was also exposed to NIR irradiation as a control. Afterward, the SOSG fluorescence intensity was measured using a multi‐mode microplate reader (Synergy HT, Biotek, VT, USA). For intracellular ROS detection, Cells seeded on 12‐well plates overnight were incubated with 500 nm NF@VER for 6 h, and then exposed to 690 nm laser irradiation (100 mW/cm^2^, 4 min). Next, the cells were washed three times with PBS and incubated with 10 µm DCFH‐DA (D1002U, UElandy, Suzhou, China) at 37°C for 20 min. Afterward, the cells were gently washed with PBS to remove the extracellular DCFH‐DA probe. Then detached using an enzyme‐free cell dissociation solution, and fluorescence signals were detected by flow cytometry (Beckman Coulter, CA, USA).

### 4.6 NF and NF@VER Uptake

Cells seeded on confocal dishes were incubated with 500 nm Cy3‐labeled NF for 1 h at 4°C or 37°C. Afterward, cells were washed three times with PBS buffer and imaged using a confocal microscope (Axio‐Imager LSM‐800, Zeiss, Germany). For flow cytometry analysis of uptake efficacy, cells were seeded on 12‐well plates overnight, then incubated with 500 nm Cy3‐ or Cy5‐labeled NF for the indicated time at 37°C. After washing three times with PBS, the cells were detached using an enzyme‐free cell dissociation solution (#13151014, Thermo Fisher, USA), followed by flow cytometry analysis (BECKMAN COULTER, USA).

### 4.7 ELISA

EA. hy926 cells were incubated with 10 ng/mL recombinant human VEGF protein (#293‐VE‐010, R&D Systems, USA) and various concentrations of NF for 12 h. The cell culture medium was collected, and the levels of VEGF in the supernatants were analyzed with ELISA kits (#EK183, Multi Sciences, Hangzhou, China), following the manufacturer's instructions. To determine VEGF levels in the mouse CNV model, retinal‐choroidal complexes were homogenized in RIPA buffer, centrifuged, and VEGF levels in the supernatant were quantified using a commercial ELISA kit (#EK283, Multi Sciences, China).

To evaluate the immunogenicity of NF@VER, serum and retinal‐choroidal complex homogenates were collected 30 days after intravitreal injection of NF or NF@VER. Levels of IL‐6 and TNF‐α were measured using ELISA kits (IL‐6: #EK206; TNF‐α: #EK282; Multi Sciences, China), following the manufacturer's protocols. Total protein content in the tissue homogenates was similarly assessed by BCA assay for normalization.

### 4.8 Live‐Cell Imaging

To visualize the lysosomal colocalization, cells seeded into a confocal plate were treated with 500 nm Cy3‐NF at 37°C for 6 h. The cells were washed twice with PBS and incubated with LysoTracker Green (#C1047S, Beyotime, Shanghai, China) at 37°C according to the instructions. After labeling with LysoTracker Green, the cells were washed twice. Then, the cell nuclei were stained with Hoechst (#33342, UElandy, Suzhou, China) for 10 min, followed by removing the Hoechst solution and washing with PBS three times. The images were captured using a CLSM confocal microscope (Axio‐Imager LSM‐800, Zeiss, Germany).

To visualize the lysosomal colocalization, cells seeded into a confocal plate were treated with 500 nm FAM‐NF@VER at 37°C for 6 h. The cells were washed twice with PBS and incubated with Mito‐tracker Red (#C1049B, Beyotime, Shanghai, China) at 37°C according to the instructions. The cells were counterstained with Hoechst for 10 min and imaged under the CLSM confocal microscope.

### 4.9 Cell Viability

Cells were plated on 96‐well plates (2.0 × 10^4^ cells per well) overnight and subjected to NIR irradiation (100 mW/cm^2^) for the indicated time points. Cell viability was assessed using a CCK‐8 kit according to the manufacturer's protocol. Alternatively, the cells were incubated with a Calcein/PI working solution (#C2015 M, Beyotime, Shanghai, China) for 30 min. Afterward, the cells were gently washed with PBS buffer and stained with Hoechst for 10 min. Images were captured using the CLSM confocal microscope.

### 4.10 Mitochondrial Membrane Potential Determination

EA. hy926 cells were seeded on confocal dishes overnight, incubated with various formulations for 4 h, and then exposed to 690 nm NIR irradiation (100 mW/cm^2^) for 4 min. The cells were then washed three times with PBS buffer and incubated with the TMRE working solution (#C2001S, Beyotime, Shanghai, China) at 37°C for 30 min. Afterward, the cells were gently washed with PBS buffer to remove extracellular TMRE. Finally, the cells were analyzed by flow cytometry.

### 4.11 Immunoblot Analysis

The proteins were boiled with sodium dodecyl sulfate (SDS) loading buffer for 10 min, separated by 10% SDS polyacrylamide gel electrophoresis (PAGE). The protein samples were transferred from the gel to nitrocellulose (NC) membrane and were incubated in 5% skimmed milk blocking buffer for 1 h. After that, the protein membranes were incubated with primary phosphorylated AKT antibodies (F1644, Sellcek, USA, 1:1000) or AKT primary antibody (F0004, Sellcek, USA, 1:1000), and anti‐rabbit horseradish peroxidase (HRP)‐secondary antibodies subsequently (UT2001, Utibody, China). The target protein was visualized by the chemiluminescent substrate and imaged with a chemiluminescence imaging system (Tanon 4800, Tanon, China).

### 4.12 Wound‐Healing Assay

EA. hy926 cells cultured in a 12‐well plate with 90% confluency were scratched by a sterile 10 µL pipette tip in the center of the cell monolayer. After washing three times with PBS, cells were treated with 10 ng/mL VEGF and the indicated NF or NF@VER, followed by NIR irradiation. The wound region was imaged, and the percentage of wound closure was analyzed using ImageJ software.

### 4.13 Tube Formation Assay

The tube formation assay was performed as previously described [40]. Briefly, EA. hy926 cells were seeded in a Matrigel‐coated 24‐well plate at a density of 2 × 10^5^ cells per well. Each well was treated with 10 ng/mL VEGF and various formulations and irradiated. After 24 h, tube formation images were captured using a light microscope (Olympus, Japan). The angiogenesis analyzer plugin of ImageJ was used to measure the total branching length and the number of branch points.

### 4.14 Transwell Assay

EA. hy926 cells suspended in serum‐free medium were seeded in the upper chamber of 24‐well transwell plates (2.5 × 10^5^ cells per well). Meanwhile, complete culture medium was added to the lower chamber. The cells were treated with various formulations as described above and 10 ng/mL VEGF, then irradiated. After 24 h of incubation, cells on the upper side of the transwell membrane were gently removed using cotton swabs. The cells that migrated downward and attached to the bottom of the lower chamber were stained with 1% crystal violet and imaged under a light microscope. The migrated cell area per field was quantified using ImageJ's particle analysis function.

### 4.15 Three‐Dimensional Sprouting Assay

The EA. hy926 cells (1 × 10^3^ cells per well) were cultured overnight in medium containing 0.24% carboxymethyl cellulose in non‐adherent round‐bottom 96‐well plates for endothelial cell sprouting assays. Spheroids were collected by centrifuging at 200 g for 15 min. Then, the spheroids were evenly seeded on Matrigel‐coated 24‐well plates. A culture medium with 10 ng/mL VEGF and various treatments was added on top of the gel. After NIR irradiation, capillary‐like sprout formation was examined using microscopy, and angiogenic activity was quantified by measuring the total sprout length.

### 4.16 Animal Experiments

C57BL/6J mice (6–8 weeks, male) were obtained from the Tianjin Medical University Animal Laboratory. All the mice were housed in conventional experimental holding areas with an alternating 12 h light/dark‐period control, regulated temperature at 21 ± 4°C, relative humidity at 50 ± 10%, and ad libitum feeding on UV‐treated food and 1 µm‐filtered water. The animal studies were conducted under the approval of the Institutional Animal Care and Use Committee of Tianjin Medical University General Hospital (ID: IRB2025‐DWFL‐676).

All animal experiments were designed with random allocation. Before grouping, all mice were numbered and randomly assigned to the control group and the experimental group using a computer‐generated random number table, ensuring that there were no significant differences in weight and genetic background among the groups at the beginning of the experiment. In the histopathological scoring and image quantitative analysis, the operators were unaware of the grouping information of the animals.

### 4.17 FFA

The mice were intraperitoneally injected with 0.2 mL of 2% fluorescein sodium after anesthesia and pupil dilation. FFA images were acquired using Heidelberg confocal retina angiography (HRA, Germany). The ImageJ software calculated each leakage area of the FFA images.

### 4.18 VEGF‐Induced Retina Vascular Leakage Model

The C57BL/6J mice were intravitreally injected with 100 ng recombinant mouse VEGF 164 protein (493‐MV, R&D Systems, USA) using a 33‐gauge beveled needle on Day 1 to establish the retina vascular leakage model. After 24 h, PBS, 2 µm NF, and 1 µl Conbercept (KANGHONG, China) were injected intravitreally for treatment. FFA was performed on Day 2 and Day 4 to analyze vascular leakage.

### 4.19 Laser‐Induced CNV Mouse Model

The laser‐induced CNV mouse model was established as previously described [41]. Briefly, an Nd: YAG laser produced four laser spots (LB‐002088 REVB, Lumeni, Beijing) around the optic disk at the 3, 6, 9, and 12 o'clock positions. The laser spots had a diameter of 100 µm, a power of 200 mW, and an exposure time of 0.15 s. The successful laser burn signal was indicated by the presence of a bubble, which signified rupture of Bruch's membrane. The anterior segment of mice was imaged with a slit‐lamp microscope (ZEISS, Germany). FFA was performed on day 5 and day 14 to analyze vascular leakage.

For in vivo NF@VER determination, the Cy5‐NF@VER was intravitreally injected into the eyes of CNV mice, and the Cy5 signals were monitored with a Syngene PXi imaging system (Synoptics Ltd, USA). After 48 h, the CNV mice were anesthetized, and the eyes were examined. The eyes were fixed, and flat‐mounted RPE‐choroid slides were stained with the anti‐CD31 antibody (1:200 dilution, MAB1398Z, Millipore‐Sigma, USA).

### 4.20 Immunofluorescence Staining

To confirm the efficacy of neovascular inhibition by the NF@VER, CNV mice with or without treatment were anesthetized. The mice's eyes were extracted on Day 14 and fixed in 4% PFA. The RPE‐choroid‐sclera complex was harvested after 2 h. Afterward, the ocular tissue was stained with anti‐CD31 antibody at 4°C overnight, followed by incubating with secondary antibodies, and imaged with the CLSM confocal microscopy. The CNV lesion area was quantitatively analyzed using ImageJ software.

### 4.21 Analyses of Vascular Leakage and Perfusion

Vascular leakage was assessed as previously described [42]. Briefly, mice were perfused via the left ventricle with warm PBS containing FITC‐conjugated dextran (FD70S, Sigma–Aldrich, USA). After 10 min of circulation, the eyes were enucleated and fixed in 4% PFA for 1 h at room temperature. The dissected retinas were then washed with PBS and subjected to sequential processing. This involved overnight permeabilization at 4°C with 1% Triton X‐100 in PBS, followed by blocking for 12 h using a solution of 5% BSA and 0.5% Triton X‐100 in PBS. Subsequently, the retinas were incubated overnight at 4°C with an anti‐CD31 antibody (1:200, MAB1398Z, Millipore‐Sigma, USA) prepared in the same blocking solution. After thorough washing, a final incubation step was performed with the corresponding secondary antibody for 2 h. Imaging was performed using a confocal fluorescence microscope (LSM980, Carl Zeiss, Germany), and the relative mean fluorescence intensity of FITC‐dextran leakage was quantified with ImageJ software.

### 4.22 Hematoxylin and Eosin (H&E) Staining

Tissue specimens from the heart, liver, spleen, lungs, and kidneys were harvested and immediately fixed in 4% paraformaldehyde (PFA), followed by paraffin embedding. Sections were prepared from the paraffin‐embedded tissues and subjected to H&E staining. Histological examination was performed using an optical microscope (OLYMPUS, Japan). Additionally, 30 days post‐intravitreal injection of NF@VER, enucleated eyeballs were collected and fixed in FAS eyeball fixative. The fixed eyeballs were embedded in paraffin, and both visually oriented and histologically structured sections were obtained. These sections were stained with H&E for further histological evaluation (OLYMPUS, Japan).

### 4.23 Statistical Analysis

All the data were presented as mean ± SD, except for specific notifications. GraphPad Prism 9.0.1 software was used for statistical analysis. A multiple paired t‐test was used to analyze the difference in the FFA leakage area of pretreatment and posttreatment. A one‐way ANOVA test combined with Dunnett's post hoc test was utilized for multi‐group analysis, whose data follows a normal distribution, while the production of ROS of free VER and NF@VER, and the cell viability of EA. hy926 cells with VER under NIR or not were analyzed by Two‐way ANOVA followed by Dunnett's multiple comparisons test and Šídák's multiple comparisons test. A nonpaired two‐tailed Student's *t*‐test was performed for the two groups’ data analysis in the colocalization of lysosomes or mitochondria, or CNV, and NF or NF@VER overlap coefficient, and PCC of colocalization. *p* < 0.05 was considered statistically significant.

## Author Contributions

S.X. and H.Y. supervised the project and designed the experiments. M.L., Y.Y., S.M., H.Z., J.S., and C.X. performed the experiments. M.L., Y.Y., S.C., Y.H., H.X., and S.W. analyzed data. M.L., S.X., and H.Y. wrote the manuscript.

## Conflicts of Interest

The authors declare no conflicts of interest.

## Supporting information




**Supporting File**: advs73707‐sup‐0001‐SuppMat.docx.

## Data Availability

Research data supporting the findings of this study are available within the paper in the main text or the Supplementary file.
