# Supplementary material for: DNA Nanoflower LYTACs Enable Efficient VEGF Degradation and Verteporfin Loading for Combined Therapy of Wet Age‐Related Macular Degeneration
Source: Adv Sci (Weinh). 2026 Jan 28;13(16):e15852. doi: 10.1002/advs.202515852 (PMC13042913; doi:10.1002/advs.202515852)
Supplement: Supplementary file 1 — Supporting File: advs73707‐sup‐0001‐SuppMat.docx. [file ADVS-13-e15852-s001.docx]

Supporting Information

**DNA Nanoflower LYTACs Enable Efficient VEGF Degradation and Verteporfin Loading for Combined Therapy of Wet Age-Related Macular Degeneration**

Mengxuan Li^1,#^, Yan Yue^1,#^, Sijin Wu^2,#^, Xinyu He^2^, Jiayi Song^1^, Shuwen Ma^1^, Haokun Zhang^1^, Chenyu Xu^3^, Song Chen^1^, Yanming Huang^4^, Songbo Xie^1,5*^, Hua Yan^1,5*^

^1^Department of Ophthalmology, Tianjin Key Laboratory of Ocular Trauma, Ministry of Education International Joint Laboratory of Ocular Diseases, Tianjin Medical University General Hospital, Tianjin 300052, China.

^2^Wisdom Lake Academy of Pharmacy, Xi'an Jiaotong-Liverpool University, Suzhou, 215028, China.

^3^School of Medicine, Nankai University, Tianjin 300071, China.

^4^Xiamen Eye Center and Eye Institute of Xiamen University, School of Medicine, Xiamen 361104, Fujian, China.

^5^The Province and Ministry Co-sponsored Collaborative Innovation Center for Medical Epigenetics, Laboratory of Molecular Ophthalmology, Tianjin Medical University, Tianjin 300070, China.

^#^These authors contributed equally to this work.

***Correspondence authors**

E-mail addresses: zyyyanhua@tmu.edu.cn (H.Y.); xiesongbo@sdnu.edu.cn (S.X.).

**Supplementary Figures**


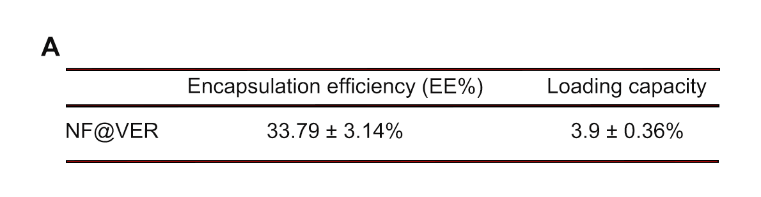


**Figure S1.** **(A)** The encapsulation efficiency (EE%) and loading capacity of VER (n=3). Data are presented as mean ± SD.


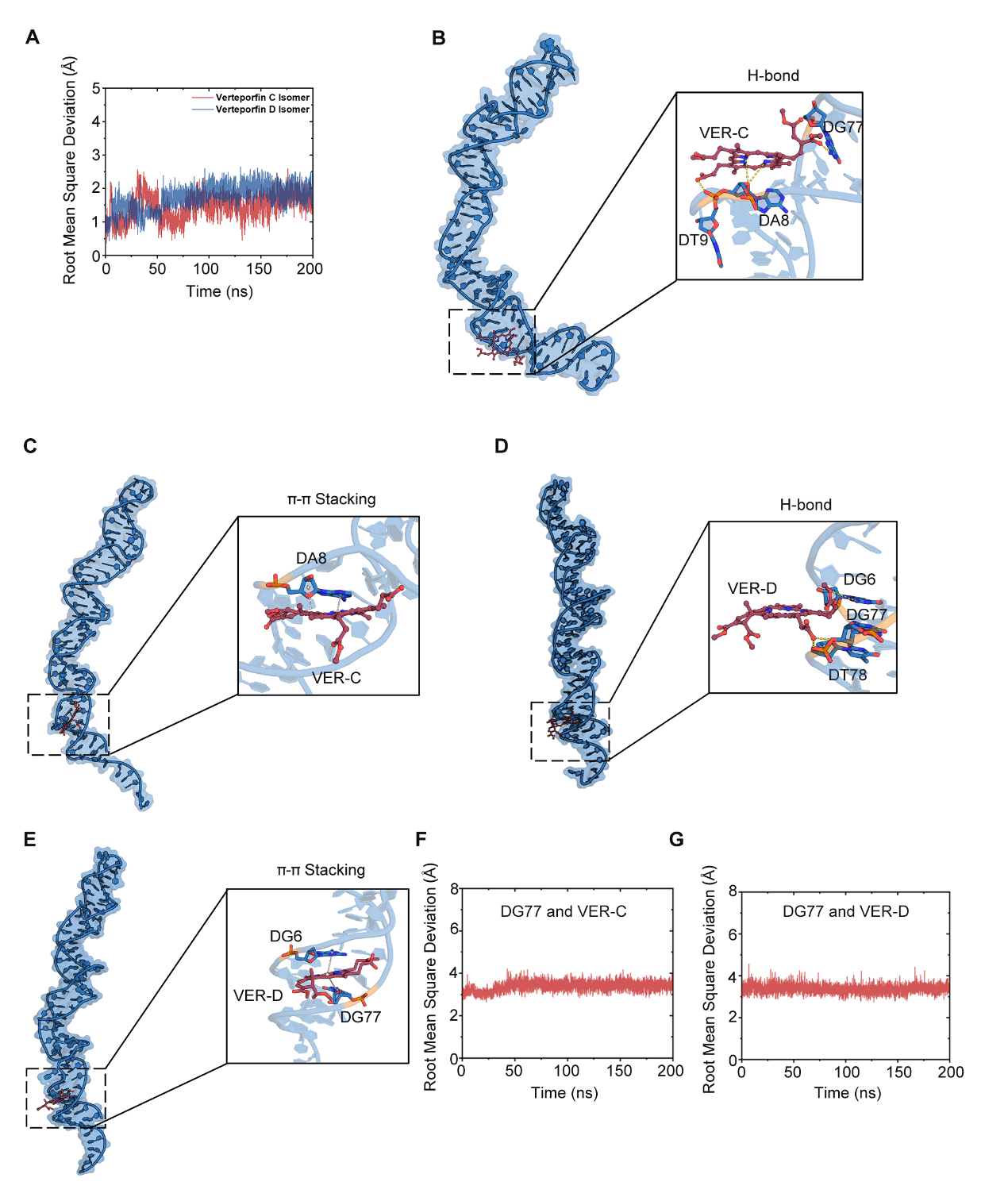


**Figure S2.** **(A)** Ligand Root Mean Square Deviation (RMSD) trajectories over 200 ns molecular dynamics (MD) simulations for VER-C isomer (red) and VER-D isomer (blue). **(B, C)** MD-revealed multi-point hydrogen bond anchoring mode (DT9, DA8, and DG77 in NF) and dual π–π stacking mode (DA8 in NF) of VER-C isomer with NF. **(D, E)** MD-revealed multi-point hydrogen bond anchoring mode (DT78, DG6, and DG77 in NF) and dual π–π stacking mode (DG6 and DG77 in NF) of VER-D isomer with NF. **(F)** Time-dependent trajectory of the minimum heavy-atom distance between DG77 and VER-C during the 200 ns MD simulation. **(G)** Time-dependent trajectory of the minimum heavy-atom distance between DG77 and VER-D during the 200 ns MD simulation.


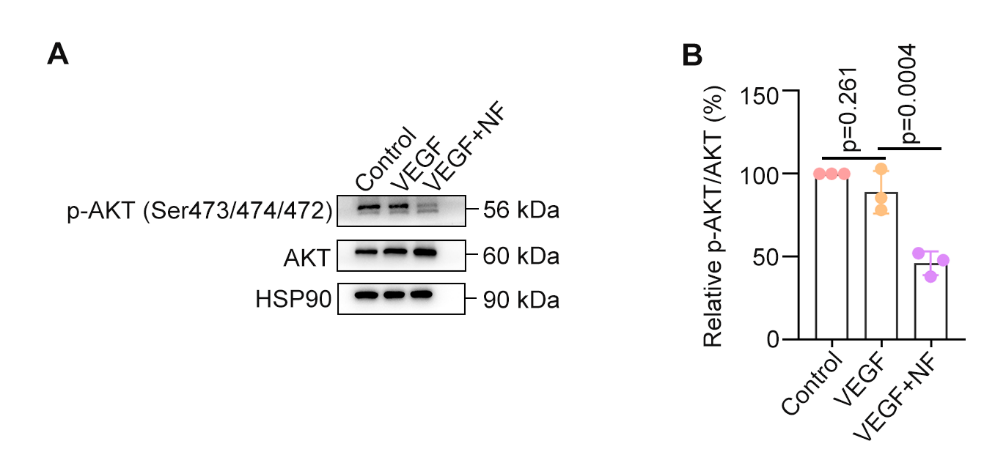


**Figure S3.** **(A, B)** NF inhibits the phosphorylation of the downstream AKT protein in VEGF-induced EA. hy926 cells (n=3). Statistical analyses were performed using One-way ANOVA followed by Tukey's multiple comparisons test. Data are presented as mean ± SD.


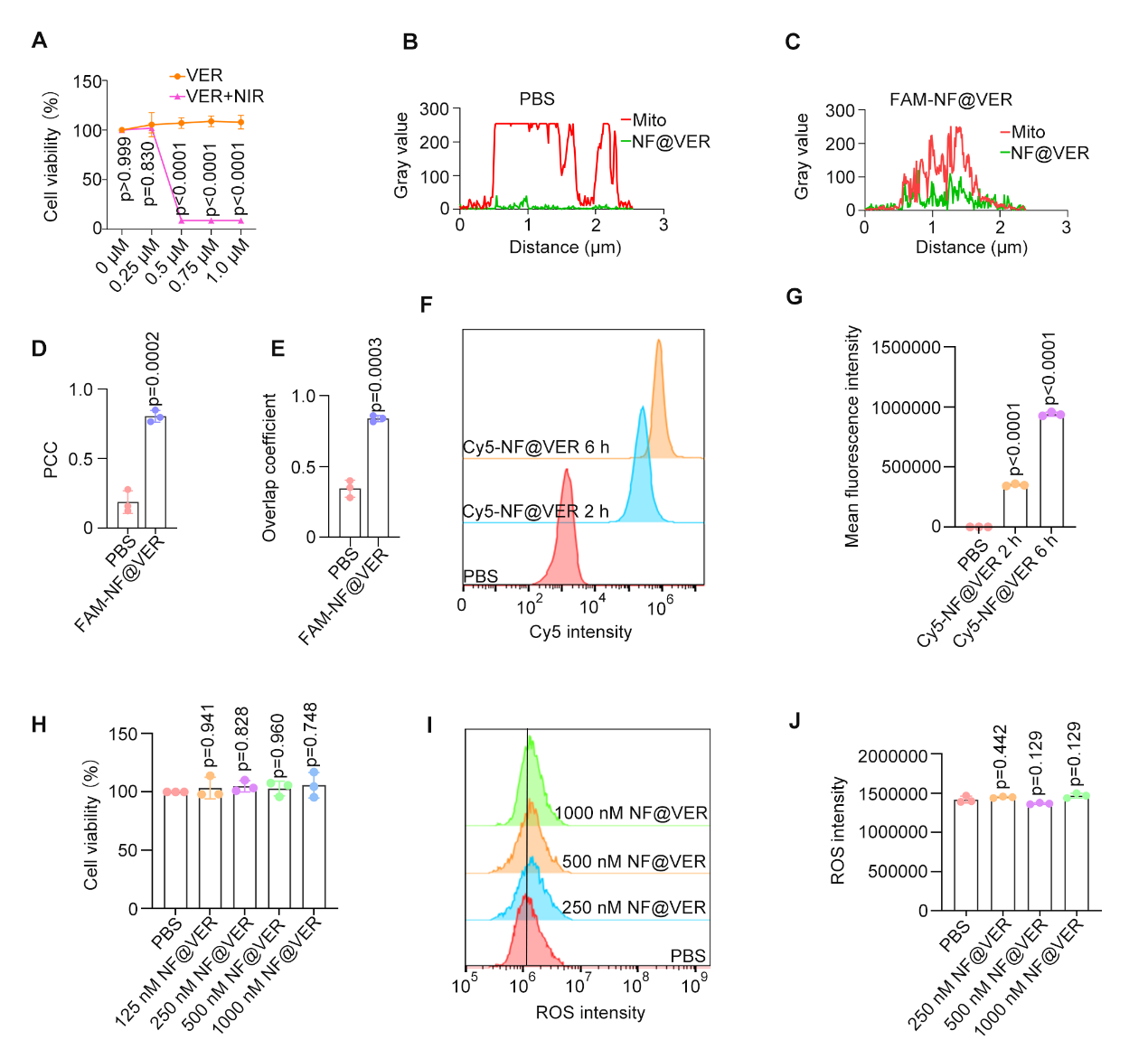


**Figure S4.** **(A)** The cell viability of EA. hy926 cells with different concentrations of VER treatment under NIR irradiation or not. **(B, C)** The colocalization of mitochondria and FAM-NF@VER. **(D, E)** The overlap coefficient (E) and Pearson correlation coefficient (D) of colocalization. **(F, G)** Flow cytometry detection (F) and quantification (G) of the internalization efficacy of 500 nM Cy5-NF@VER in ARPE19 cells. **(H)** Cell viability determination of ARPE19 cells after the treatment with various concentrations of NF@VER. **(I, J)** Flow cytometry detection (I) and quantification (J) of intracellular ROS generation after the treatment of various concentrations of NF@VER without NIR irradiation. Statistical analyses were performed using Two-way ANOVA followed by Šídák's multiple comparisons test (A) and One-way ANOVA followed by Tukey's multiple comparisons tests (D, E, G, H, J). Data are presented as mean ± SD.


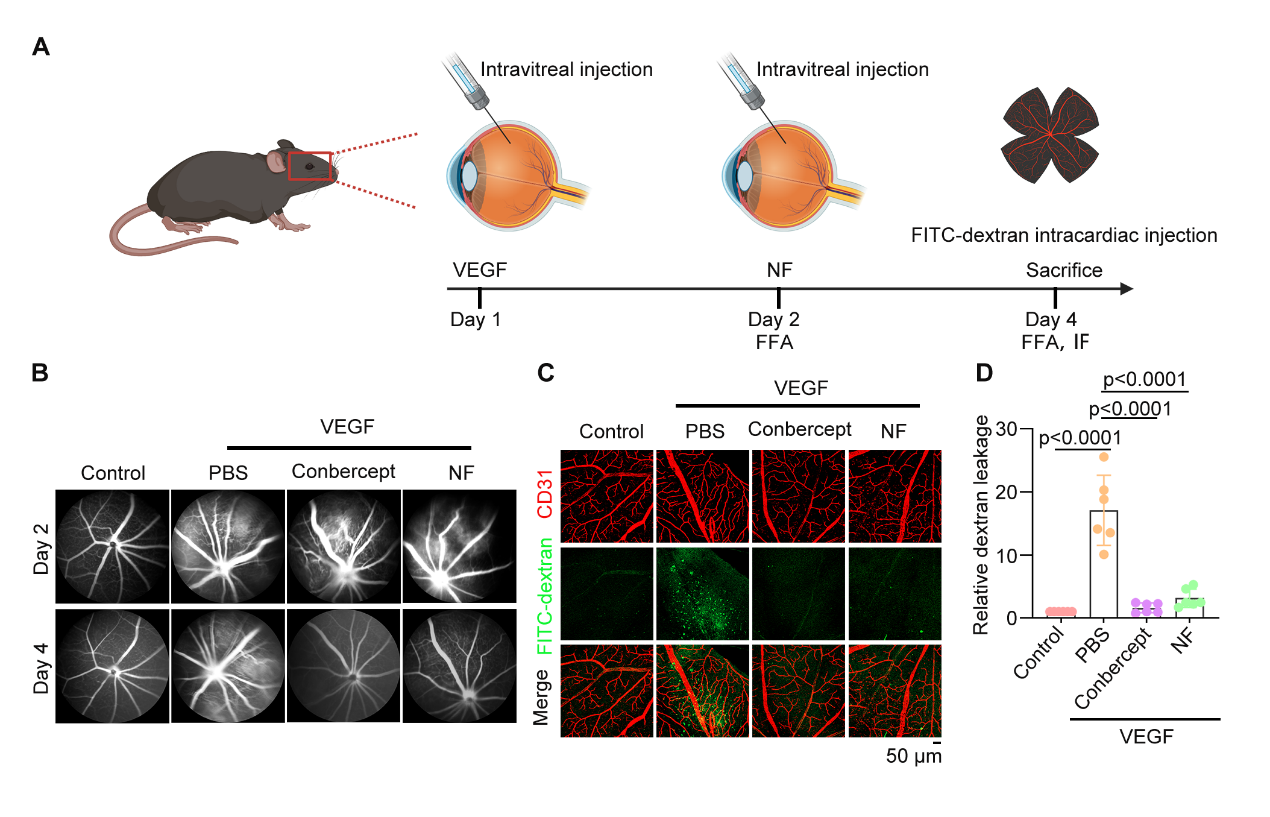
**Figure S5.** **NF suppresses VEGF-induced vascular leakage *in vivo.***

**(A)** Schematic depicting the post-treatment procedure in the VEGF-induced vascular leakage model. 100 ng VEGF was intravitreally injected to induce vascular leakage in the retina. After 24 h, PBS, Conbercept, and 2 μM NF were intravitreally injected in adult wild-type C57BL/6J mice (Created with BioRender.com). **(B)** Representative FFA images on Day 2 and Day 4. **(C, D)** Representative images of flat-mounted retina (C) showing extravasated FITC-dextran and CD31^+^ vessels and quantification of dextran leakage (D) (n = 6). Statistical analyses were performed using One-way ANOVA followed by Tukey's multiple comparisons test (D). Data are presented as mean ± SD.

**Table S1. The oligonucleotide sequences used in this study**

| Name | Sequence (5’-3’) |
| --- | --- |
| Apt^IGF2R^ | GACGCCGGGTTGGGTGGGTGGGAAGGGCGTCGCTCCCTCTTCG |
| Apt^VEGF^ | GGCCCGTATGGTGGGTGTGCTGGCC |
| LYTAC | GGCCCGTATGGTGGGTGTGCTGGCCTTTTTTTTTTACGCCGGGTTGGGTGGGTGGGAAGGGCGTCGCTCCCTCTTCGTTTTTTTTTT |
| RCA template | P-ACCCACCATACGGGCCAAAAAAAAAACGAAGAGGGAGCGACGCCCTTCCCACCCACCCAACCCGGCGTAAAAAAAAAAGGCCAGCAC |
| RCA primer | TATGGTGGGTGTGCTGGCCT |
